# Stretchable fabric generates electric power from woven thermoelectric fibers

**DOI:** 10.1038/s41467-020-14399-6

**Published:** 2020-01-29

**Authors:** Tingting Sun, Beiying Zhou, Qi Zheng, Lianjun Wang, Wan Jiang, Gerald Jeffrey Snyder

**Affiliations:** 1 State Key Laboratory for Modification of Chemical Fibers and Polymer Materials, College of Materials Science and Engineering, Donghua University, Shanghai, China; 2 Engineering Research Center of Advanced Glasses Manufacturing Technolog, Ministry of Education, Donghua University, Shanghai, China; 3 Department of Materials Science and Engineering, Northwestern University, Evanston, IL USA

**Keywords:** Devices for energy harvesting, Thermoelectric devices and materials, Electrical and electronic engineering, Thermoelectrics, Energy efficiency

## Abstract

Assembling thermoelectric modules into fabric to harvest energy from body heat could one day power multitudinous wearable electronics. However, the invalid 2D architecture of fabric limits the application in thermoelectrics. Here, we make the valid thermoelectric fabric woven out of thermoelectric fibers producing an unobtrusive working thermoelectric module. Alternately doped carbon nanotube fibers wrapped with acrylic fibers are woven into π-type thermoelectric modules. Utilizing elasticity originating from interlocked thermoelectric modules, stretchable 3D thermoelectric generators without substrate can be made to enable sufficient alignment with the heat flow direction. The textile generator shows a peak power density of 70 mWm^−2^ for a temperature difference of 44 K and excellent stretchability (~80% strain) with no output degradation. The compatibility between body movement and sustained power supply is further displayed. The generators described here are true textiles, proving active thermoelectrics can be woven into various fabric architectures for sensing, energy harvesting, or thermal management.

## Abstract (web summary)

Despite recent advances in flexible thermoelectric generators for wearable devices, current designs are unable to efficiently harvest heat flowing from human body. Here, the authors report high thermoelectric performance and stretchability in interlocked fiber-based modules for wearable devices.

## Introduction

With the escalating use of portable electronics, wearable thermoelectric generators (TEGs) are highly desired as a potential^1^ power supply to harvest electricity from human body heat^2,3^. Intense effort has been devoted to develop flexible TE devices over the past few decades. Existing TE devices are too rigid and bulky to be easily incorporated into a garment, while flexible prototype devices using an out-of-plane thermal gradient are at the moment too fragile for practical application^4^.

Despite the advances^5,6^ in output power of flexible TEGs based on carbon nanotube (CNT), poly(3,4-ethylenedioxythiophene) (PEDOT), polyaniline, or bismuth telluride (BiTe based) in recent years, the technology still faces a significant challenge for use as a wearable power supply because a good matching between TE modules and effective thermal gradient direction is required. Conventional flexible TEGs are typically assembled by arranging, folding, or stacking serial sets of tiled films including a substrate^7^, which is essentially a flat, two-dimensional (2D) architecture that merely harvests the thermal energy in the in-plane direction but poorly fit to the perpendicular temperature gradient formed between the human body and the environment. Such architecture will not work effectively as a wearable thermal harvester.

The scalable solution for a wearable thermal harvester has become a research frontier for wearable TEGs. Some pioneering works utilize a wave-like shaped polymer substrate^8^ to hold thin film-based TE materials upright in a practical direction for the heat flow. Others use island-like inorganic or metal TE materials pressed into a soft fabric with an adhesive^9^, electrochemical deposition^10^, or screen printing technique^11^ to satisfy the demand of flexibility considering the thermal gradient. Recently, a helical architecture^4,12^ is employed to change a 2D flat film into a three-dimensional (3D) architecture. However, these architectural solutions have disadvantages: the flexibility is limited because of the inorganic TE material, and the substrate to support the TE modules in the vertical direction causes parasitic loss of temperature difference which will depress the final electrical power.

Fabric could ensure the flexibility of TE devices, but 2D architecture^13,14^ is difficult to harvest heat flowing from human body to environment. Some reported works use 3D spacer fabric substrate^15^ or thick textiles substrate^16^ to hold fiber-based TE modules in 3D space. Woven TE textiles^17,18^ based on TE yarns have been described using insulating yarns to separate hot and cold side or provide a locating point for the p/n junction in the heat flow direction. However, these 3D architectures supported with substrates are not easily incorporated into a garment on a large scale. More importantly, it is extremely hard to achieve conformality and stretchability with the proposed weave patterns without sacrificing TE performance. In fact, a truly wearable, woven TEG has not been reported. Thus, the key challenge for practical application of wearable TEGs is how to fulfill the following requirements simultaneously: i) first and foremost, the TEG architecture must efficiently capture heat in the direction of the thermal gradient to provide high power density^4,10,18^; ii) TE modules possess stable and high performance, particularly its n-type segment; iii) high flexibility to enable sufficient thermal contact with body tissues of arbitrary geometry^19^; iv) many microfabricated p/n junctions per area in compact design for sufficient voltage; v) scalable architecture^20^ design to unobtrusively collect heat over a wide area to provide higher power; vi) stretchable and conformal architecture^21^ of overall device design to allow body movement without performance degradation; and vii) architecture enables the intended thermal insulation of the textile while at the same time facilitates heat transfer through the thermoelectric generator^22,23^.

Herein, we present a device engineering solution integrated with high-performance fiber-based modules that addresses all the above challenges. To this end, we first develop a π-type carbon nanotube fiber-based module using oleamine doping combined with electrospray technology. Then these TE modules are interlocked alternately in a pattern designed to successfully woven into a 3D TEGs without substrates, which distinguishes it from reported textile-based TE devices. The TE modules are self-standing to match the direction of heat flow and have excellent stretchability (~80% strain). Finally, integrating the high TE performance π-type modules into the logical architecture design contributes to a superior power density of 70 mWm^−2^ at 44 K, which is the highest output reported for a flexible organic TEG. More importantly, it is the first time TE modules made by weaving into truly stretchable textiles compatible with body movements, instead of embedding them into clothes and avoids sacrificing flexibility and output power. The device engineering in this work is compatible with any fiber active materials, and can maximize the practicality and efficiency of wearable TEGs.

## Results

### Design and performance of TE modules

**Fig. 1:**
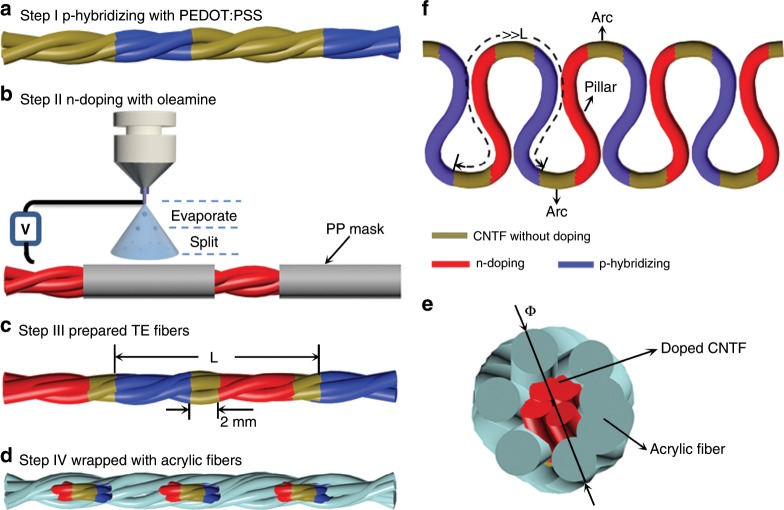
Schematic illustration of the fabrication process of the TE loops. **a** p-Hybridized CNTF. **b** n-Doped CNTF using an electrospray technique. **c** TE fibers. **d** TE fibers wrapped with acrylic fibers. **e** Cross sectional view for the structure of **d**, *Φ* is the diameter of TE fiber after being wrapped. **f** TE loops.

In this work, a case study of an active TE material of carbon nanotube fibers (CNTF) is developed. The CNTF used in this paper was prepared by twisting four CNT films synthesized by a floating catalyst method^24^, aiming at enhancing the tensile strength for subsequential weaving. It can be seen from the transmission electron microscope (TEM) and field emission scanning electron microscope (FE-SEM) image (Supplementary Fig. 1a, b) that the CNTF was ~280 µm in diameter composed of thousands of multiwalled CNTs with ~11 nm diameter. Figure 1a–f schematically exhibited the fabrication process of TE modules. First, CNTF was p-hybridized by dipping into a commercial poly(3,4-ethylenedioxythiophene):poly(styrenesulfonate) (PEDOT:PSS) solution (details is described in Supplementary Note 1). Subsequently, n-type CNTF was achieved in an equal interval using polypropylene (PP) mask by oleamine doping combined with electrospray technology. Therefore, a TE fiber with alternatively doped n- or p-segment at the distance of (*L* − 4 mm)/2 was formed, as schematically shown in Fig. 1c, where *L* (mm) is the length of p–n repeat unit of the fiber. The 2 mm long undoped section is electrically conducting and treated as an electrical interconnection (electrode) between the two doped sections. Furthermore, to avoid a short circuit, the doped CNTF was wrapped with acrylic fibers using a coverspun technique, as displayed in Fig. 1d, e, except the electrode segment, which was exposed to maximize the thermal contact between the TEG and the human body (details can be seen in Supplementary Fig. 2). Finally, the doped TE fibers were bent into TE loops (TE modules) with repeat length *L* using a knitting needle of the required diameter 3 or 6 mm, following a path composed of circular and straight parts, here named arc and pillar, respectively, as illustrated in Fig. 1f. It should be noted that the electrode and p/n leg must be located in the arc and pillar position of a TE loop, respectively, for the fabrication of a TEG. The mechanical stability of a TE loop was further studied by measuring the change of electrical resistance versus bending angle and bending cycles. As shown in Supplementary Fig. 3a–c, the variations of electrical resistance are <0.5%, indicating the TE loop is mechanically stable.

**Fig. 2:**
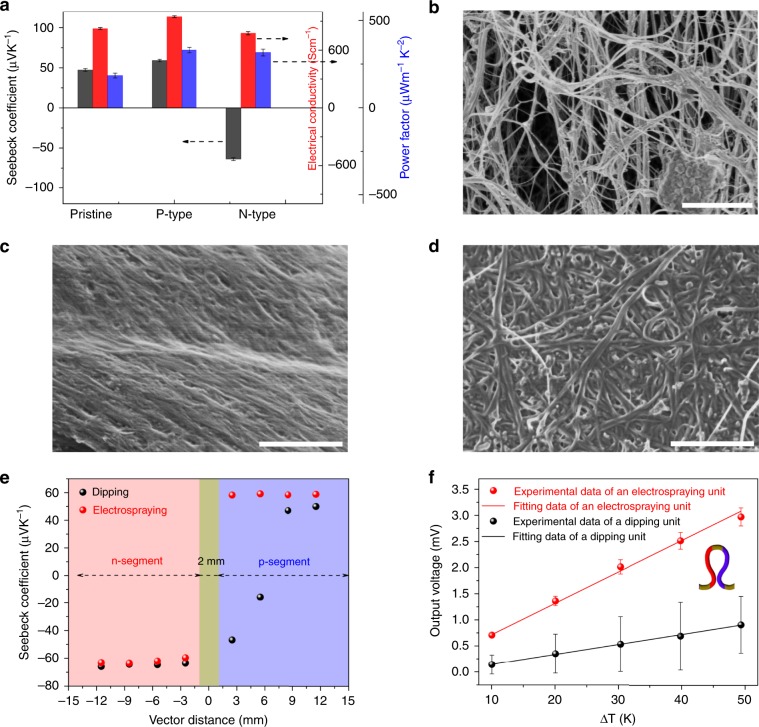
Performance of CNTF. **a** TE performance of pristine CNTF, doped CNTF, and hybridized CNTF. **b**–**d** FE-SEM images of the surface of CNTF. **b** Pristine CNTF, **c** p-hybridized CNTF, and **d** n-doped CNTF. Scale bars, 1 μm. **e** Seebeck coefficient versus vector distance of a TE unit. The repeat length is set to 3.2 cm, and the center of electrode is served as the origin. **f** The output voltage of the two TE units studied in **e**. All error bars represent s.e.m.

The TE material performance plays a significant role on properties of TE devices. As depicted in Fig. 2a, pristine CNTF possess a positive Seebeck coefficient (*α*) owing to oxygen impurity^25–28^, suggesting hole-like majority carriers. The electrical conductivity of CNTF increases from 820 to 950 Scm^−1^ after p-hybridizing, combining with an increase in *α* (as previously reported in CNT/PEDOT:PSS composites^29^), resulting in an enhancement of power factor from 185 to 330 µWm^−1^K^−2^. Comparing the surface morphology of pristine and p-hybridized CNTF in Fig. 2b, c, a vaguely visible fibrous network arising from the coating layer of PEDOT:PSS can be observed after the dipping process. The presence of PEDOT:PSS layer was further confirmed by Raman spectrum in Supplementary Fig. 4.

It remains a great challenge to obtain stable n-type CNT based TE devices. CNTF was converted into an n-type material (Fig. 2a) with a Seebeck coefficient of −64 µVK^−1^ after being doped with oleamine for 1 h based on electrospray technology (doping condition is manifested in Supplementary Fig. 5a, b). Excitingly, the Seebeck coefficient is stable after being exposed to air for over 800 h (according to Supplementary Fig. 6), and this stability is better than recent work on n-type CNT^25,30^. In contrast with traditional polyethyleneimine (PEI) n-doping method^26,31^, oleamine doping combined with electrospray technology is superior in the respect of short doping time to ensure high Seebeck coefficient, as well as excellent air stability. The coating of oleamine on the CNT can be seen in Fig. 2d, which leads to thicker bundles of CNT than pristine CNT. The insulated oleamine coating in turn results in a slight decrease in electrical conductivity, nevertheless, a high power factor of 320 µWm^−1^K^−2^ was achieved owing to the high Seebeck coefficient. Raman spectra in Supplementary Fig. 7 provide further insight into the n-doping effect, in which the enhanced intensity ratio of D to G mode illustrates the covalent as well as non-covalent interaction^26^ between the CNT and oleamine produced using electrospray technology.

As is known, to integrate both n- and p-segments into a single π-type CNT unit is a great challenge due to the infiltration of dopants into the neighboring parts^16,32^. We compared our electrospray doping method with the conventional dipping method, as shown in Fig. 2e. A negative Seebeck coefficient appears in a part of the p-segment, in the case of the dipping method. The electrospray doping process is shown in Supplementary Fig. 8. The droplet containing the dopant was split into monodispersed micro droplets driven by repulsive electric charges, and then deposited at an accelerated speed because of the electrostatic force. Finally, the accelerated droplets of oleamine quickly infiltrated the CNT instead of diffusing in the horizontal direction. As a result, a 3-fold rise in output voltage per repeat unit was obtained by electrospray technology due to precise n-doping localization as shown in Fig. 2e, f. Note that electrospray doping is not compatible with all dopants.

### Effective configuration of TE loops for TEGs

**Fig. 3:**
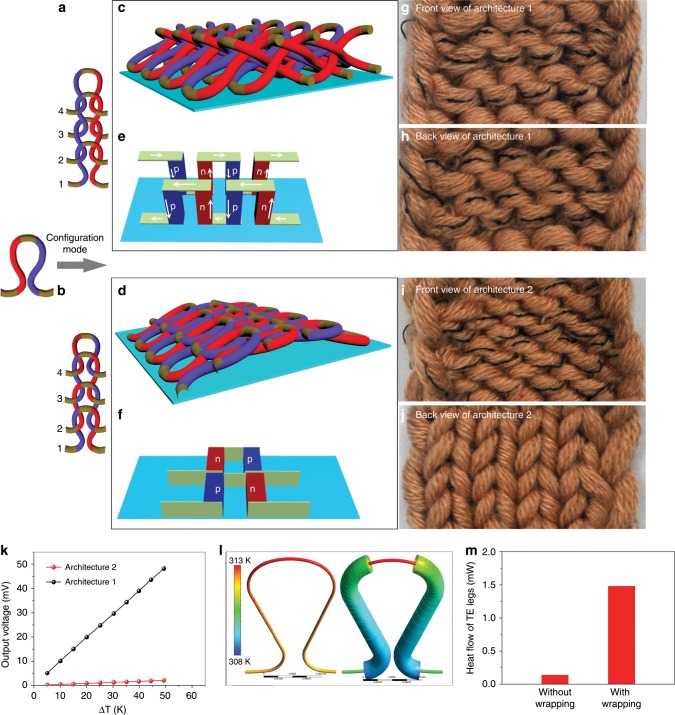
Architecture and thermal performance of TE textiles. **a**, **b** Configuration modes of TE loops, **b** shows the conventional modes**. c**, **d** Schematic illustrations of TE textiles configured by mode **a** and **b**, respectively. **e**, **f** Illustrations of TE modules corresponding to the 3D TE devices **c** and conventional ones **d**, respectively. **g**, **h** Photographs of architecture with 15 units displayed in illustrations **c** (*L* = 32 mm, *Φ* = 3 mm). **g** shows the front view, and **h** shows the back view. **i**, **j** Photographs of architecture with 15 units displayed in illustrations **d** (*L* = 32 mm, *Φ* = 3 mm). **i** shows the front view, and **j** shows the back view. **k** Output voltage of two architectures versus temperature difference. Black and red circle represent architecture 1 and 2, respectively. Simulations of **l** temperature distribution and **m** heat flow along the loops (TE legs) with or without wrapping.

**Fig. 4:**
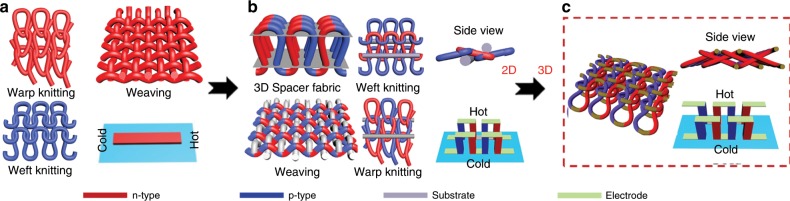
Comparison with other textile-based mode for TEGs. **a** 2D in-plane TEGs, including warp knitted, woven, and weft knitted fabric. **b** 3D out-of-plane TEGs supported with substrates, including warp knitted, woven, weft knitted, and 3D spacer fabric with supporting yarns or substrates. **c** 3D out-of-plane TEGs without substrate fabricated using elasticity of bending fibers interlocked in 2D space.

High TE efficiency is essential for a TEGs. However, another primary challenge in the wearable TE devices is the design of effective architecture that matches the direction of heat flow, which is in the out-of-plane direction. In order to form a 3D architecture to satisfy the above requirement, the prepared TE loops should be interlocked as displayed in Fig. 3a. As indicated, the pillar position of TE loop 1 is covered with the arc position of TE loop 2, whose pillar position covers the arc position of TE loop 3, forming an alternately interlocking mode. Most interestingly, owing to the elastic force of bending fibers, TE loops interlocked in the above mode could automatically stand at an angle along the out-of-plane direction without a substrate. The rows of this weave can form a 3D architecture to make a TE textile, as schematically exhibited in Fig. 3c. The angle is determined by the weaving parameters (the repeat length *L*, the diameter *Φ,* etc). Note that this interlock mode can be achieved in large area fabrics in the textile industry. Figure 3e shows the illustrations of TE modules corresponding to the conventional TE devices for Fig. 3c, and it proves a compact and effective TE modules that p/n legs are connected electrically in series and thermally in parallel. Here, a TE textile with 15 TE units is fabricated to provide more details of the formation of the 3D architecture in Fig. 3g, h. As can be seen, only the exposed electrode locating in arc position of every TE unit can be seen on the both surfaces, which benefits the thermal contact with the hot or cold source. However, the acrylic fiber-wrapped TE legs are not visible, because the wrapped TE legs are inside the textiles, which is testified by the cross section optical microscopy image along the longitudinal direction (Supplementary Fig. 9a–d). Note that only the interlock mode in Fig. 3a can lead to the formation of a 3D architecture, utilizing elasticity of the bending fibers. For comparison, Fig. 3b shows conventional configuration mode that the pillar position of TE loop is covered with the arc position of the next TE loop successively. This interlock mode results in the formation of a 2D architecture in the in-plane direction as illustrated in Fig. 3d, f. Photographs of TE textiles with 15 TE units in Fig. 3i, j further denotes that all the exposed electrodes are shown on one surface, while the TE legs wrapped with fibers lie flat on the other surface. The output voltages of the two architectures were further compared in Fig. 3k, showing the output voltage of 3D architecture is ~24 times higher than that of 2D architecture. As indicated above, just by changing the interlock mode, a conventional 2D TE device can be changed into a 3D architecture and leads to high performance. In addition, the performance of the directly woven 3D TE unit was also compared with a CNT unit with just TE fibers through this 3D textile substrate in Supplementary Fig. 10 and Supplementary Movies 1 and 2. As is shown, directly woven 3D TE units possessed higher temperature difference and output voltage. Detailed analysis can be seen in Supplementary Fig. 10. In comparison with other textile-based mode for TEGs (shown in Fig. 4), the automatically formed 3D architecture owing to the elastic force rather than a supporting substrate is a key difference as opposed to the reported 3D TE devices, where mechanical properties, stability, and TE performance could be limited by the substrate.

In actual application, the hot end temperature of TEG is dependent on the contact with human body surface, while the cold end relies on ambient convection. Therefore, thermal design in the electricity harvesting is vital, especially for wearable applications. On the basis of basic thermoelectric theory^4,33^, the electric power (*P*) converted from heat for a wearable TE harvester can be described as follows: *P* = *η*_*0*_Δ*T*_TE_*Q*_TE_ (details refer to Supplementary Note 2), where *η*_*0*_ is almost a constant value in the case of given TE materials with intrinsic figure of merit *zT*. *Q*_TE_ and Δ*T*_TE_ represent the heat flowing through and temperature difference across the TE legs, respectively. Hence, the electricity-generating ability of a TE device requires both a Δ*T*_TE_ and *Q*_TE_. TE devices prepared from folded 2D films on a substrate must take the reduced *Q*_TE_ into consideration, which is originated from thermal shunting through the substrate^22^. In contrast, the automatically standing TE modules in this paper can contact with the heat source/cold side directly without substrate although the insulating wrapping may have a similar effect. To assess the effect of wrapping layer on the thermal performance, the temperature difference Δ*T*_TE_ and heat *Q*_TE_ through the standing TE legs are simulated using finite element analysis (FEA). Details of simulation are given in the Method section. The numerical simulation results in Fig. 3l, m denote a slightly higher temperature difference and a 10-fold rise in heat flowing through TE legs in comparison with the TE loops without wrapping. As further shown in Supplementary Fig. 10, higher and more stable temperature difference (dynamic detection can be seen in Supplementary Movies 2 and 3) can be maintained by this wrapped unit, as compared with an unwrapped one, which also contributes to a higher output voltage. This result corresponds to the numerical simulation results. The above results can be explained by the typical thermal circuit (Supplementary Note 2)^33^. The combined thermal resistance (*Θ*_HX_), whose inverse represents the ability of heat exchange in hot/cold side, connects that of the TE materials (*Θ*_TE_) in series. Based on a given temperature difference between heat source and environment (Δ*T*_supply_), *Q*_TE_ can be obtained by the equation: *Q*_TE_ = *Q*_HX_ = Δ*T*_supply_/(*Θ*_HX_ + *Θ*_TE_). Actually, the wrapping layers can enlarge the area for heat dissipation, that is, the thermal resistance is depressed in the cold side. Thus, the reduced *Θ*_HX_ contributes to an enhanced *Q*_TE_, followed by the increased Δ*T*_TE_ according to the equation: Δ*T*_TE_ = *Θ*_TE_*Q*_TE_. It implies that the wrapping layers can not only avoid a short circuit, but also optimize the thermal design of the TE devices, indicating the rationality of our design for wearable TEGs.

### Biaxial stretchability with stable TE performance

**Fig. 5:**
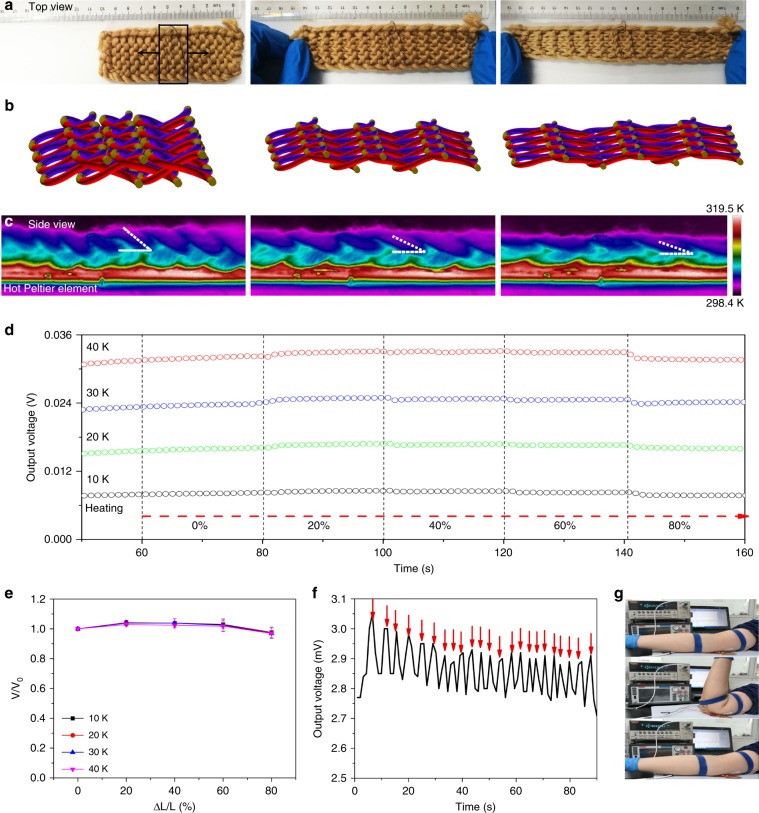
Longitudinal stretchability of the TE devices. Photographs of **a** the top view and **b** schematic after longitudinal stretching by 0%, 40%, and 80%. The highlight area in **a** indicates the effective TE modules with 15 (3 × 5) units, whose strain is equal to the overall textile. **c** Infrared thermal images of the side view contacted with a Peltier element (~318.1 K). The dashed line in **c** marks the standing angle of the TE loops. **d** Curve of the real-time output voltage responses of the TE device stretched to 80% under different temperature differences. The temperature difference is created by two Peltier elements. **e** TE performance degradation versus longitudinal strain. Average values of at least six measurements are taken. All error bars represent s.e.m. *V*_0_ is the output voltage of TE device without stretching. **f** Plot of the real-time output voltage responses of TE devices attached to a moving elbow. **g** Photos show the elbow movement corresponding to plot **f**. The red arrows in plot **f** point to the bending elbow movement shown in **g**.

In contrast to previously reported 3D architecture TE devices with limited stretchability^10,11,16,18,32^, the TE textiles in this paper are not only flexible but also stretchable. More importantly, the longitudinal stretchability of this TE device mainly results from the configuration of each TE unit, instead of a deformation of each TE unit reported in flexible/stretchable TE devices previously^4,12,34^. Not actually flexing the TE material during stretching avoids the potential of fracturing the TE materials. As indicated in Fig. 5a, the TE devices can be stretched in the longitudinal direction by over 80%. Fig. 5b schematically exhibits the corresponding structural change during stretching by 80%: automatically the aggregative TE loops are drawn apart, but just very slight deformation are produced in each TE unit, following a 50% decrease in standing angle from ~40° in initial status to ~20° in strain of 80%, as shown in Fig. 5c. Thus, the excellent stretchability actually takes advantage of the elastic force originating from interlock mode shown in Fig. 3a. Stretching and automatically aggregating are a pair of reversible operations, which gives the TE devices outstanding conformality, as displayed in Supplementary Movie 4. Supplementary Fig. 11a indicated that stretching the device over 80% does not cause electrical failure. Figure 5d, e further exhibited the TE performance degradation versus longitudinal stretching at various temperature difference created by two Peltier elements. Small strain of 20% could immediately lead to a ~3% increase in output voltage, which can be ascribed to the enhanced thermal contact resulting from a flatter interface on the edge of TE devices with the hot Peltier element as shown in Fig. 5c. In the case of further stretching, decreased Δ*T*_TE_ caused by decrement in standing angle (smaller standing angle enhances thermal convection with heat source) predominates, inducing a negative effect on the output voltage. Nevertheless, the maximal decrement obtained in the case of 80% strain and 40 K is <3.2%, suggesting the potential of the fabricated device working as a stretchable and wearable power generator.

The other advantage of the TE device is its biaxial stretchable property rather than the current stretchable TE devices that is uniaxial^4,34^. As displayed in Supplementary Fig. 12a–c, the TE devices can be transversely stretched over 60%, accompanied by sustained slight increase in output voltage, which can reach ~10%. The increasing output voltage could attribute to a larger contact area with heat source during transverse stretching.

We further verified the practicality of our TE devices by examining the compatibility with body parts of arbitrary geometry or body movement and output stability. The TE devices with 15 TE units were attached to human elbow and endured the strain resulting from elbow movement. As can be seen in Fig. 5f, g, a voltage of ~2.8 mV was generated when TE modules were attached to a human elbow and a ~4% amplitude in voltage attributing to the stretch caused by elbow movement could be sustainably obtained during dynamic testing procedure (this dynamic testing is shown in Supplementary Movie 5). To the best of our knowledge, this is the first time a stretchable TE device, which is a truly wearable textile fabric comparable to commercial clothes is prepared. This result indicates the ability of our architecture design to alleviate various problems in wearable thermoelectric fields, as opposed to conventional flexible TE devices, such as stretchability, conformality, large area, and stability to continually harvest electricity from human body.

### TEG output optimization

Power density described as the output power per occupied area has been an effective evaluation index for wearable TE devices according to previous reports^18,20^. As analyzed in Supplementary Note 3 and Supplementary Fig. 13, the power of our TE devices increased first and then decreased with enhancing repeat length *L*, which is consistent with the traditional rigid TE devices taking contact effect into consideration^35^, and the maximum is obtained at *L* = (*Θ*_HX_*λS* + 4 *N*)/*N*. For the occupied area, TE loops can be assumed as undeformed uniform cylinders maintaining contact at the crossing points referring to conventional fabric^36^. The loop shape built based on this simplified assumption is displayed in Supplementary Fig. 14a, b, and it can be seen that the occupied area can be estimated as Area ≈ 4*NΦ*^2^, here *N* is the total unit amount, and *Φ* is the diameter of TE fiber after being wrapped (details is analyzed in Supplementary Fig. 14). However, the quadratic relationship between occupied area and diameter *Φ* does not mean that a simple reduction in *Φ* will contribute to higher power density. It should be noted that smaller diameter *Φ* and longer TE repeat length *L* will cause a loose TE textile, followed by a decrease in the standing angle of each TE loop, which has a detrimental effect on heat flowing as well as mechanical properties of the TE device. Here, we introduce the concept of an unfilling factor *δ* = *L/Φ* (ref. ^37^) to numerically characterize the tightness. To fabric a TE textile with optimized power density and mechanical properties, a high tightness *δ* should be given at first step. The constant *δ* contributes to a *L* depending *Φ*, indicating the significance of *L* for power density optimization.

**Fig. 6:**
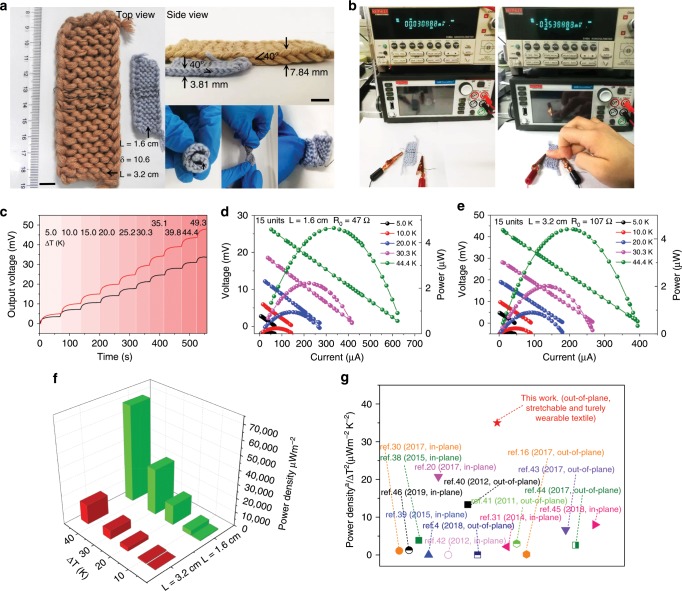
Output performance of the woven fabric TEG. **a** Photograph of TEG composed of 15 units (3 × 5) with different *L* and identical *δ*. The parameters of the larger one is set to *L* = 32 mm, *Φ* = 3 mm, while that of the smaller one is set to *L* = 16 mm, *Φ* = 1.5 mm. Scale bars, 1 cm. **b** The output voltage display of TEG (15 units) with repeat length of 16 mm before and after fingertip touching. **c** The output voltage contrast of the two TEGs at various steady temperature differences given by Peltier elements. **d**, **e** Power output of the TEGs with *L* of 16 mm/32 mm at various steady temperature differences. *R*_0_ is the internal resistance of TEGs. **f** Power density of the TEGs with different repeat length *L* at various steady temperature differences. **g** Maximum power density normalized to temperature difference squared Δ*T*^2^ of our device compared with several literature data points. The power density^a^ shown here is expressed as the output power per unit occupied area, details of calculation are displayed in Supplementary Table 1.

A high tightness *δ* of 10.6 is given and the TE repeat length *L* of TEG with the same amount of units was adjusted from 32 to 16 mm to optimize power density. A dramatically decreased occupied area can be seen in Fig. 6a. Besides, the thickness of the TEG reduced from 7.8 to 3.8 mm, but the ~40° standing angles of TE legs were maintained. Figure 6a also shows the ability of the prepared TEG to adapt to various loading conditions, e.g., bending, twisting, and folding. Furthermore, a 3.5 mV voltage can be immediately detected when human fingertip touches the small TEG as shown in Fig. 6b. The output voltage at various steady temperature differences are displayed in Fig. 6c. When a through thickness temperature difference is applied, an obvious Seebeck voltage is immediately created. After reducing the repeat length to 16 mm, the voltage decreases by ~26%, in accordance with the positive dependence of temperature difference across TE legs Δ*T*_TE_ on repeat length *L* (Supplementary Note 3). Conversely, the power increases after reducing the repeat length to 16 mm as shown in Fig. 6d, e. Using 44.4 K temperature difference, the power enhances from 4.40 to 4.64 μW, accompanying an short-circuit current increasing from ~450 to ~700 μA. The results indicate that reducing internal resistance *R*_0_ (from ~107 to ~47 Ω) can recoup the power losses caused by reducing output voltage when repeat length is adjusted from 32 to 16 mm. A 4-fold rise in power density is obtained after reducing the repeat length from 32 to 16 mm as exhibited in Fig. 6f. Giving 44.4 K temperature difference, the power density increases from 14,000 μWm^−2^ with *L* = 32 mm to 69,000 μWm^−2^ with *L* = 16 mm.

Since power is proportional to the temperature difference squared as shown in Supplementary Note 3 and Supplementary Fig. 15, it is logical to carry out a comparison of output performance based on power density normalized to temperature difference squared Δ*T*^2^, taking the diversity of the given temperature difference studied in different reports into consideration. The output performance (35 μWm^−2^K^−2^) of our TEG with 16 mm repeat length (*δ* = 10.6) is superior to previously reported flexible organic TEGs^4,16,20,30–32,38–45^ and even some inorganic generators^46^, as shown in Fig. 6g (details of calculation is displayed in Supplementary Table 1). More importantly, our TEG can be easily woven into apparel in a large scale rivaling commercial clothes owing to its excellent flexibility and wearability. On the basis of output performance for 16 mm repeat length, a TE apparel covering ~40 % surface area of an adult (0.86 m^2^, ~190,000 couples in series) can generate ~200 μW power and ~20 V open circuit voltage in 2.6 K temperature difference across TE textiles, resulting from human body (26 °C ambient) as shown in Supplementary Fig. 16, which is expected to be applied in powering microwatt wearable body sensors, such as electrocardiogram^47^ and pulse oximeter^48^, especially in extreme conditions. The power consumption of such miniature device is hundreds of μW.

The above results verify the practical prospects of our TEGs in powering portable electronics. The power density of TEG can be improved by further appropriately changing both the repeat length and thickness of wrapping layers, owing to complex repeat length *L* and diameter *Φ* dependence of power, occupied area and combined thermal resistance. It is easy to wrap the TE fibers with an ultrathin insulating layer using electrospinning technique if a short repeat length is needed. Furthermore, this architectural design is applicable to a wide range of fiber-based active materials, such as metal wires, fibers covered by active materials using drop-casting, dip coating, screen printing, and magnetron sputtering techniques. The use of more efficient inorganic-based fibers could further enable an even higher power density. In addition, the interlock mode of TE loops in this work can be achieved in large area in textile industry, opening opportunities for the forthcoming industrial production. These certify the applicability of our TEG design in electricity supply by body heat.

## Discussion

In conclusion, alternately interlocked TE loops can enable thermal resistance matching as well as excellent stretchability with slight performance degradation. The TE modules are directly woven into textiles, instead of embedding them into clothes. A truly wearable TEG rivaling commercial cloth is prepared. Integrating the oleamine electrospray doping method and logical architecture design leads to high TE performance of π-type modules with a power density of 70 mWm^−2^ at 44 K, which is superior to previous organic TEG. The compatibility with body movement and the superior power density demonstrates the application prospect. Furthermore, we provide multiple possibilities to further enhance the power density. The concepts indicated in this paper could also be applied in other fiber-based active materials.

## Methods

### Materials

CNTF utilized in the whole experiment is obtained by twisting four CNT films (consolidated by water bath) purchased from JieDi Nano, Incin China. On the basis of manufacturer’s specifications, CNT films are synthesized by a floating catalyst method^24^ with width of ~900 μm. PEDOT:PSS with PH 1000 grades is purchased from H.C. Starck and oleamine (80–90%) is purchased from Aladdin. All chemicals were used as received without any further purification.

### Fabrication of doped CNTF

Based on the desirable repeat length *L* of final TE loops, the length of p/n and electrode (undoped) segment are designed to (*L* − 4 mm)/2 and 2 mm, respectively. To distinguish p- and n-segment, a tiny knot is tied at the electrode location, which has no effect on the electrical conductivity. For p-hybridized segments, CNTF passes through a succession of PEDOT:PSS bath with width of (*L* − 4 mm)/2 in an equal interval, followed by dipping in the bath for 12 h. Subsequently, the p-hybridized segments are washed by deionized water carefully, followed by drying at 60 °C in air ambience for 4 h. For further n-doping process based on electrospray technique, CNTF, where PP film served as masks covers both the p-hybridized and electrode segments, is connected to the ground to act as a collector. The electrospraying solution is prepared by dissolving 4.2 g oleamine in 20 mL absolute ethanol under stirring at room temperature. The as-prepared solution is fed through a plastic syringe assemble with a 27 G (internal diameter 0.4 mm) stainless steel needle by a peristaltic pump at a constant feed rate of 0.1 mLh^−1^. The distance between the needle tip and CNTF collector in Fig. 1b was 7 cm and a high voltage of 24 kV was applied to the solution to electrospray micro/nano droplets out of a needle tip. The electrospraying process was carried out at a relative humidity of ~35% with 10 cm spinneret width and 1 cms^−1^ spinneret speed for 1 h. Subsequently, the excess layer stockpiled on the CNTF surface owing to electrospraying was easily wiped off with airlaid paper.

### FEA

Commercial software of ANSYS is employed to study the temperature field and heat flow across TE legs, when one end of the TE unit is in contact with hot side of 313 K and the other end is exposed to air at 298 K. Assuming that TE unit with length *L* = 32 mm consists of two semicircles with 3.25 mm radius and two straight segments. There is no contact thermal resistance between wrapping layer and CNTF surface, and the heat transfer coefficient at wrapping layer surface is set to be 5 Wm^−2^K^−1^. The thermal conductivity of CNTF and wrapping layer is 26 and 0.051 Wm^−1^K^−1^, respectively.

### Characterization

The micro morphology of CNTF and CNT is conducted using FE-SEM (su8000, Hitachi) and field emission TEM (JEM-2100F, JEOL). The Raman spectra (inVia-Reflex, Renishaw) with a 633 nm laser are recorded to analyze the p-hybridizing and n-doping effect. The TE performance of prepared CNTF in this paper is measured in air at room temperature. The electrical conductivity *σ* (Scm^−1^) is calculated as follow: *σ* = *l*/*RS*, here *l*, *R*, and *S* is the length (cm), electrical resistance (Ω), and cross section area (cm^2^) of CNTF, respectively. This measurement approach for *σ* is commonly used in wire-based materials^32,49^. *R* is measured using a four-terminal method (Keithley 2450, 5806 attachment) to eliminate the influence of lead wire resistance, combining with silver connecters and paste (schematic see Supplementary Fig. 17a) to reduce contact resistance. *S* is calculated under the assumption that the cross section of CNTF is circular, and its diameter is measured by FE-SEM and Nano measurer software. Seebeck coefficient *α* is measured by a homemade equipment based on the equation *α* = −Δ*V*/Δ*T*, here potential differences Δ*V* arising from eight temperature differences Δ*T* was recorded by Keithley 2182 A (measurement details can be seen in Supplementary Fig. 17b). The linear correlation (*R*^2^) between Δ*V* and Δ*T* should be >0.999. Test items were tested at 12 times of 6 samples for an average value. Power was measured by a homemade system shown in Supplementary Fig. 18.

## Supplementary information

Supplementary Information

Description of Additional Supplementary Files

Supplementary Movie 1

Supplementary Movie 2

Supplementary Movie 3

Supplementary Movie 4

Supplementary Movie 5

## Data Availability

The data that support the findings of this study are available from the corresponding author upon reasonable request.
