# Supplementary material for: Stretchable fabric generates electric power from woven thermoelectric fibers
Source: Nat Commun. 2020 Jan 29;11:572. doi: 10.1038/s41467-020-14399-6 (PMC6989526; doi:10.1038/s41467-020-14399-6)
Supplement: Supplementary file 1 — Supplementary Information [file 41467_2020_14399_MOESM1_ESM.pdf]

Stretchable fabric generates electric power from woven thermoelectric fibers

Tingting Sun[1], Beiying Zhou[1], Qi Zheng[1], Lianjun Wang[1★], Wan Jiang[1,2★], Gerald Jeffrey Snyder[3★]

This PDF file includes:

Supplementary Figure 1
Supplementary Figure 2
Supplementary Figure 3
Supplementary Figure 4
Supplementary Figure 5
Supplementary Figure 6
Supplementary Figure 7
Supplementary Figure 8
Supplementary Figure 9
Supplementary Figure 10
Supplementary Figure 11
Supplementary Figure 12
Supplementary Figure 13
Supplementary Figure 14
Supplementary Figure 15
Supplementary Figure 16
Supplementary Figure 17
Supplementary Figure 18

Supplementary Table 1

Supplementary Note 1
Supplementary Note 2
Supplementary Note 3

Supplementary References

[1]State Key Laboratory for Modification of Chemical Fibers and Polymer Materials, College of Materials Science and Engineering, Donghua University, Shanghai, China. [2]School of Material Science and Engineering, Jingdezhen Ceramic Institute, Jingdezhen, China. [3]Department of Materials Science and Engineering, Northwestern University, Evanston, USA. ★e-mail: wanglj@dhu.edu.cn; wanjiang@dhu.edu.cn; jeff.snyder@northwestern.edu

Supplementary Figures

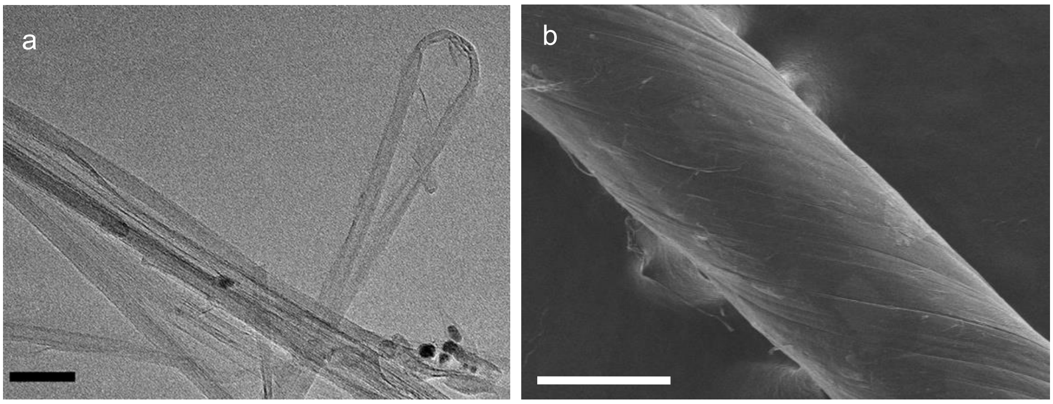

Supplementary Figure 1 | Morphology of the CNTF. a, HR-TEM image of the CNTs in the CNTF. Scale bars, 50nm. **b**, FE-SEM image of a CNTF. Scale bars, 200μm.

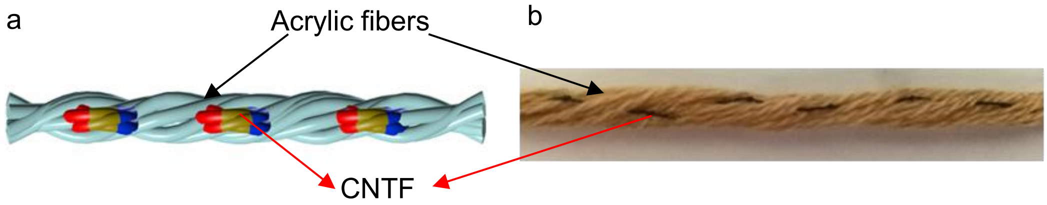

Supplementary Figure 2 | Morphology of wrapped TE fibers. (**a**) Schematic and (**b**) Photographs of doped CNTF wrapped with acrylic fibers.

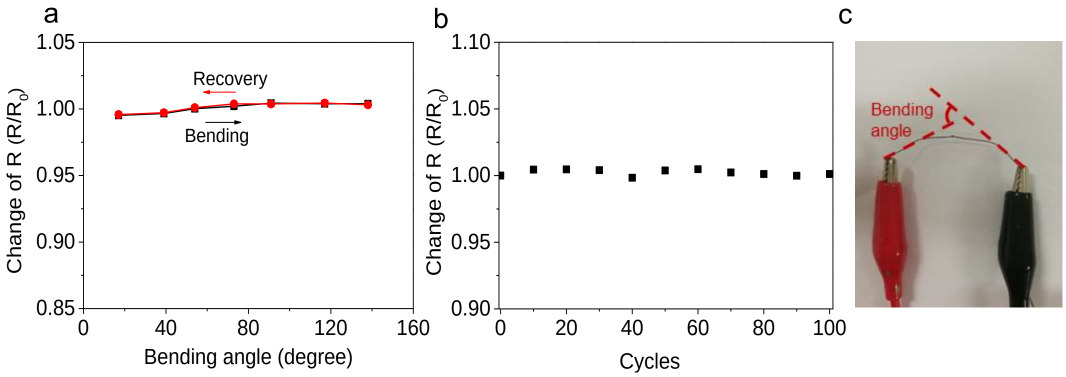

Supplementary Figure 3 | Electrical resistance stability of TE loops. Change of electrical resistance (R/R_0) versus (**a**) bending angle and (**b**) cycles at bending angle of 91°**.** R_0 is the electrical resistance of CNTF before being bent into loops. **c,** Illustration of bending angle.

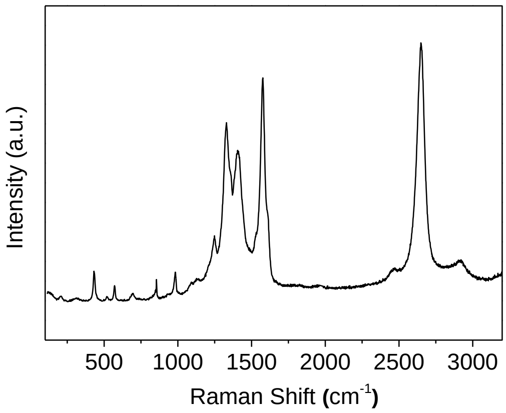

Supplementary Figure 4 | Raman spectrum of p-hybridized CNTF.

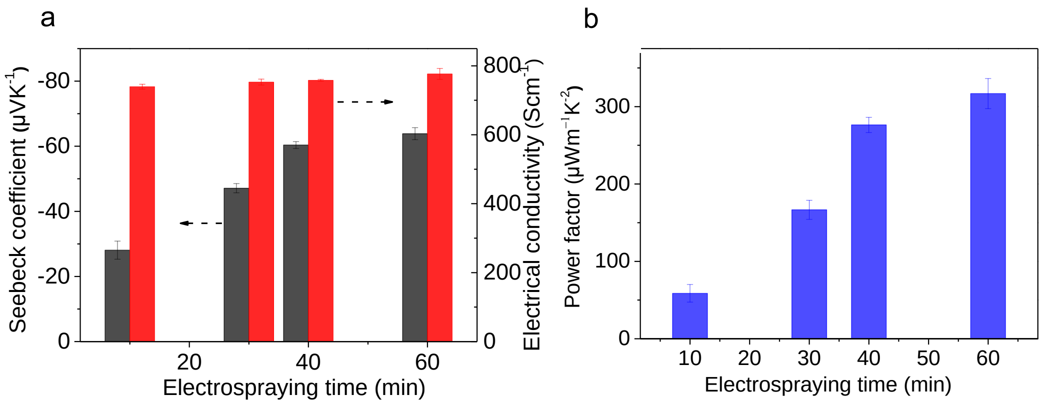

Supplementary Figure 5 | TE performance of n-doped CNTF versus electrospraying time. a, Seebeck coefficient and electrical conductivity. **b**, Power factor.

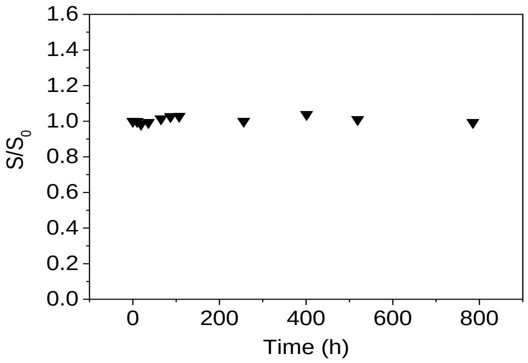

Supplementary Figure 6 | The stability test of the CNTF doped with OA for 1h.

The Supplementary Figure 6 shows good stability of OA doping CNTF, which can be attributed to three factors: (1) OA Itself, giving example that OA usually serves as a ligand for stabilizing quantum dot structures due to its stability[1]. (2)The π-π conjugation between OA unsaturated bonds and CNT, as well as the similar polarity, enhances the interaction between CNT and dopants. (3) Electrospray as a method of doping, should ensure uniformity.

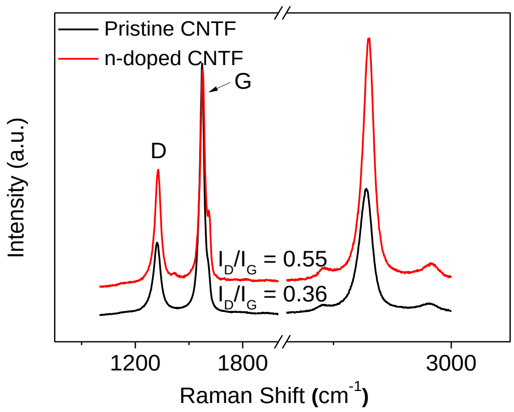

Supplementary Figure 7 | The Raman spectra of CNTF. The black curve shows the raman spectra of pristine CNTF, the red one represents the CNTF which is doped with OA for 1h based on electrospray technology.

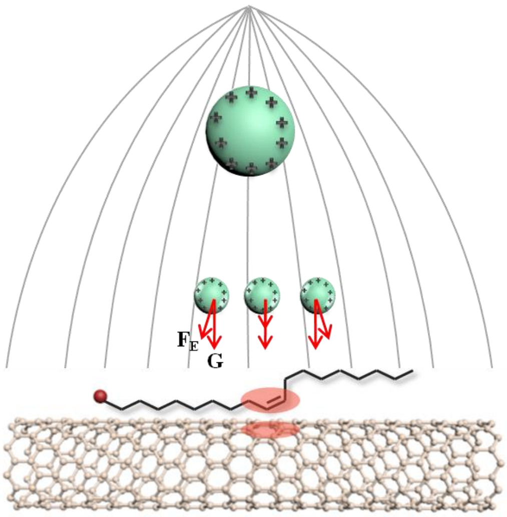

Supplementary Figure 8 | Schematic illustration of the electrospraying and doping process. . F_E is the electrostatic force acting on the charged droplet. G is the gravity force. The π-π conjugation between OA unsaturated bonds and CNT, as well as and similar polarity, enhances the interaction between CNT and dopants.

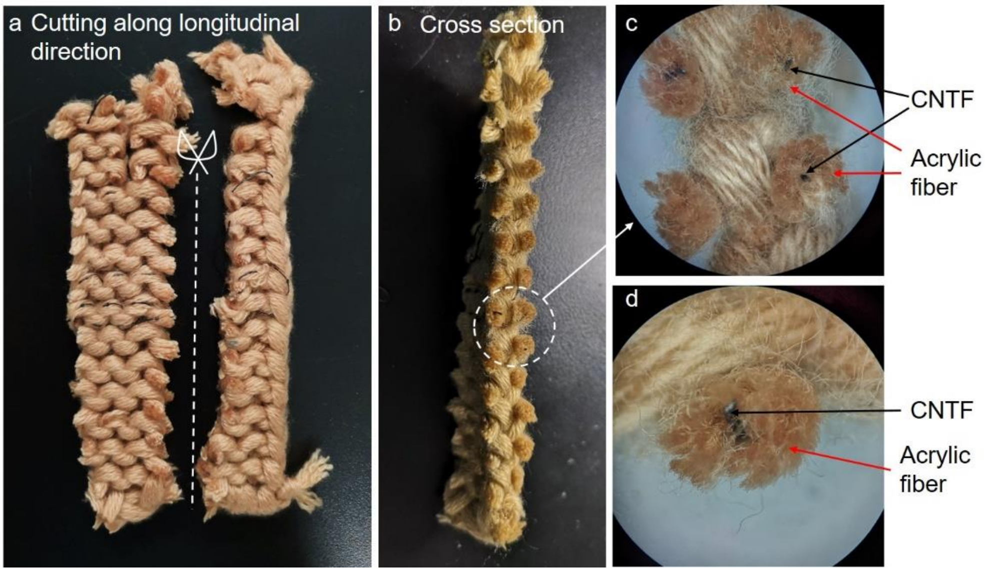

Supplementary Figure 9 | Cross-section optical microscopy image of the TE device. a, Cut of the device along longitudinal direction. **b,** Photographs of the cross section in (**a**). **c,d,** Optical microscopy image for the highlighted area in (**b**).

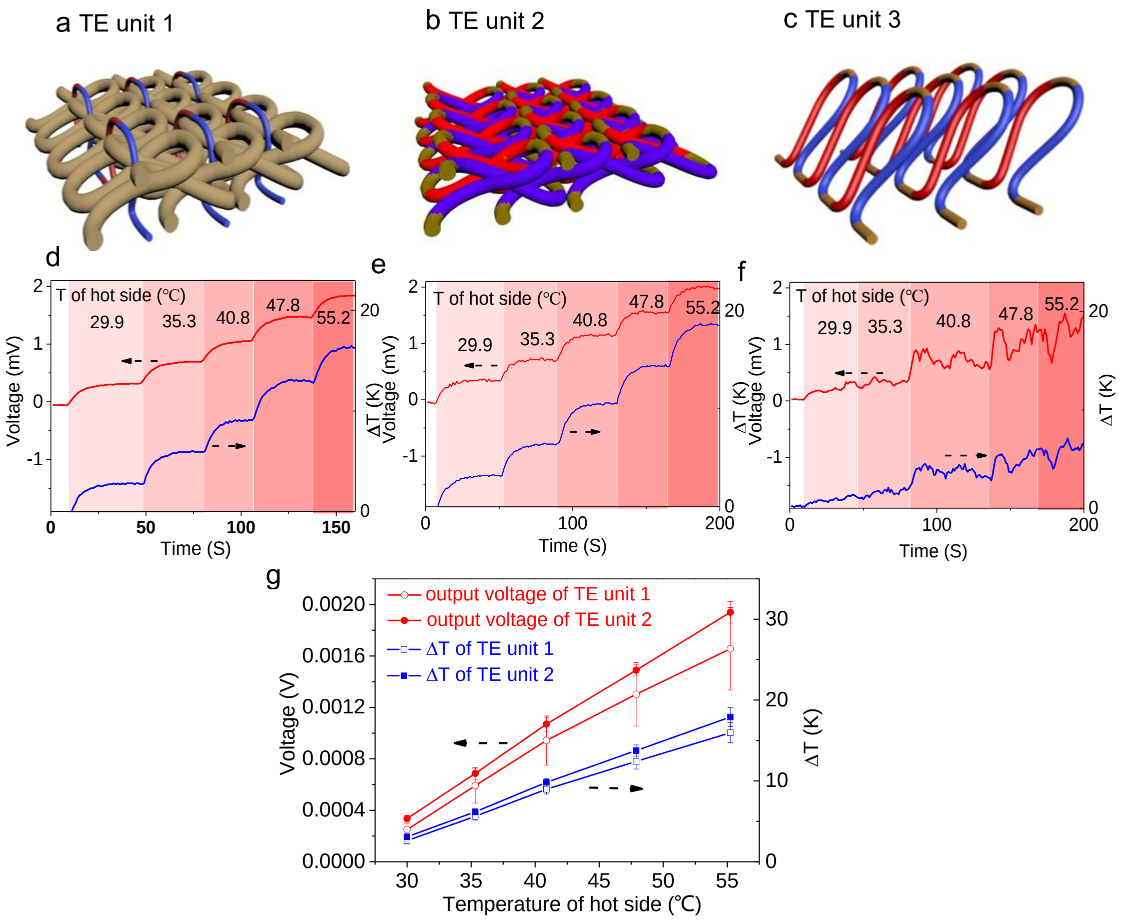

Supplementary Figure 10 | Structure and performance contrasts between three type TE units. a, Schematic illustrations of TE units that just pass the CNT TE loop through the 3D textiles

substrate. **b,** Schematic illustrations of TE units directly woven by wrapped CNT TE loop in this work. **c,** Schematic illustrations of TE units composed of un-wrapped CNT TE loop. **d,e,f** temperature difference and corresponding output voltage change of TE unit (**a**),(**b**) and (**c**),respectively, when one end of these units are contact with hot side at different temperature and the other end is exposed to air at 298K. In this way, the heat flows from the hot side to the environment, which is consistent with the practical heat flow direction during the wearing. These results display the performance of one unit. The temperature difference between the hot plate and the TE loop end exposed to air is detected with infrared thermal imager. The corresponding dynamic detection of temperature change can be seen in Supplementary Movies1-3. **g,** output voltage and temperature difference of unit 1,2 versus hot plate temperature. Test items in (**g**) were tested at 9-10 times of 3 samples for an average value.

In the contrast between TE unit 1 and 2, the TE leg length of unit 1 is determined by the thickness of the substrate, which results in a lower temperature difference and output voltage as shown in Supplementary Figure 10g. More importantly, the un-interlocked TE loops in unit 1 is difficult to be fixed inside, changing their forms easily. In addition, the TE loops in unit 1 has no stretchability as the substrate does. These indicate the rationality of our design (directly woven 3D TE units) for wearable TE generators.

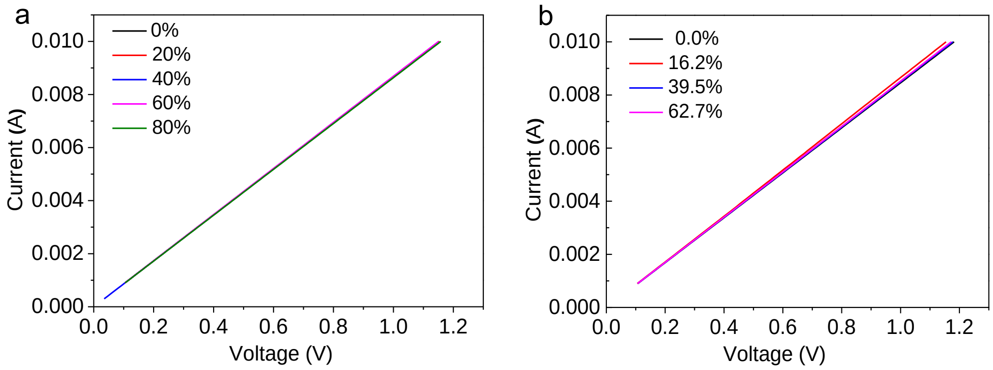

Supplementary Figure 11 | Electrical stability of the TE devices. Electrical stability versus longitudinal (**a**) and transverse (**b**) stretching of different strain.

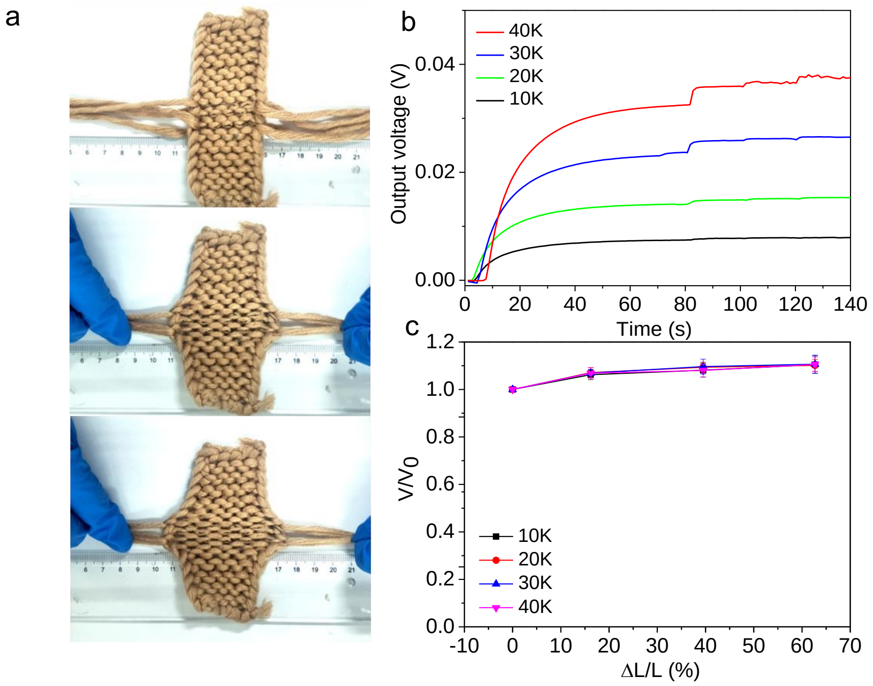

Supplementary Figure 12 | Transverse stretchability of the TE devices. a, Photographs of the top view after transverse stretching by 0%, 39.5% and 62.7%. **b**, Curve of the real-time output voltage responses of the TE device transverse stretched to 62.7% under different temperature difference. The temperature difference is created by two Peltier elements. **c**, TE performance degradation versus transverse stretching. Average values of at least 6 measurements are taken. V_0 is the output voltage of TE device without stretching force. All error bars represent s.e.m.

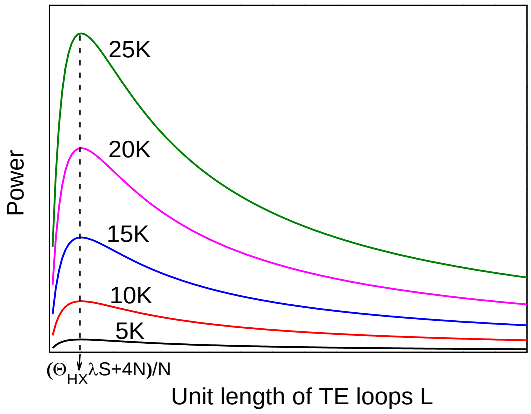

Supplementary Figure 13 | The trend chart of the power depending on the repeat length L. Detailed analysis can be seen in Supplementary Note 3.

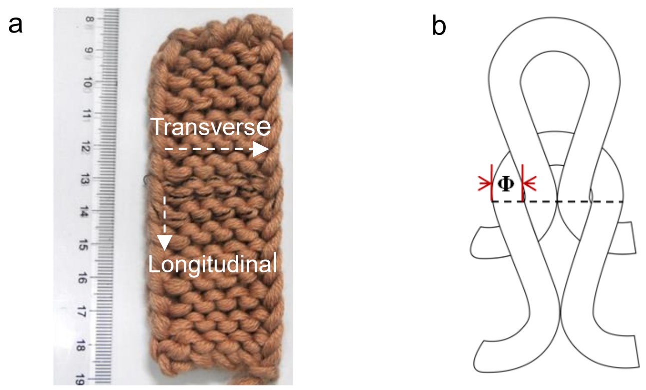

Supplementary Figure 14 | Estimate of occupied area. a, A case of a TE device with 15 units (3x5 TE loops, L = 32 mm, Φ = 3 mm). **b**, Loop shape based on simplifying assumption. As shown in Supplementary Figure.14a, the occupied area can be served as a rectangle. The length of the rectangle along the longitudinal direction l_l can be expressed as $l_l \approx N_l\Phi$. The length of the rectangle along the transverse direction l_t can be written as $l_t \approx N_t l_p$, here N_l /N_t is the unit amount along the longitudinal/transverse direction, l_p is the projection length of the arc of a TE loop on the plane. As exhibited in Supplementary Figure.14b, based on simplifying assumption, l_p can be estimated as $l_p \approx 4\Phi$. Thus, the occupied area can be expressed as Area $\approx 4N\Phi^2$, here N is the total unit amount.

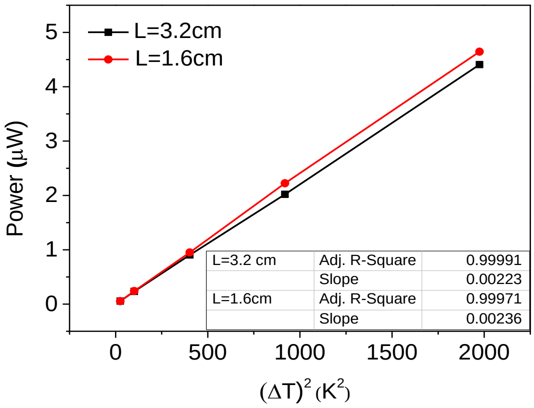

Supplementary Figure 15 | Relationship between power and temperature difference squared. Linear correlation (R^2), which is higher than 0.999 in the case of unit length of 16 mm or 32 mm, indicates the good proportional relationship between power and temperature difference squared.

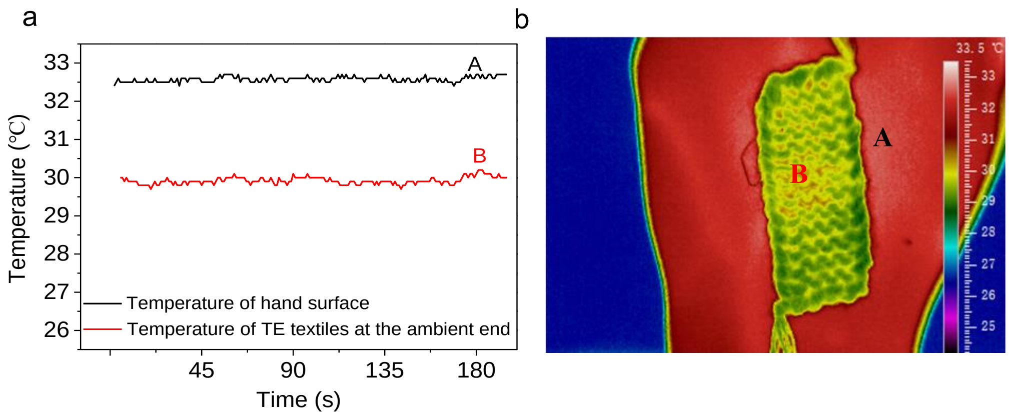

Supplementary Figure 16 | Temperature of hand surface and cold end of the TE units. a, The recorded data in point A and B corresponding to the infrared thermal images (**b**). Point A shows the temperature change of the hand surface, B displays that of the cold end of the TE devices. The repeat length L is 16mm, the cold end of TE units is exposed to ambience at 26℃.

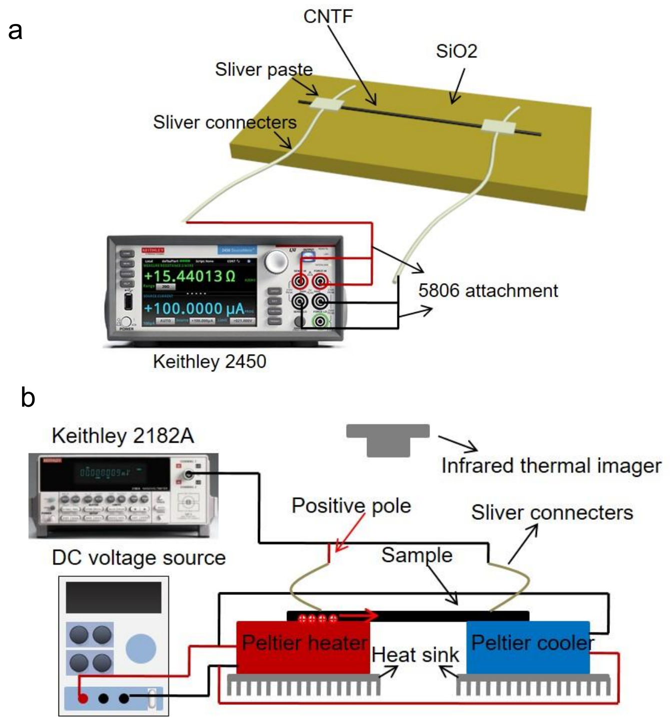

Supplementary Figure 17 | Illustrations of TE performance mearsurements. The schematic of (**a**) electrical conductivity σ measurement and (**b**) seebeck coefficient measurement. For seebeck coefficient measurement, the temperature difference is obtained from two Peltier elements driven by DC voltage source (CE0030050T, Rainworm). The heat sink is used to maintain stable temperature in hot or cold side. Besides, the value of temperature difference is recorded by an infrared thermal imager (FOTRIC 226). The probe point is set in the outside close to the junctions of CNTF and sliver connecters (diameter is 250 μm).

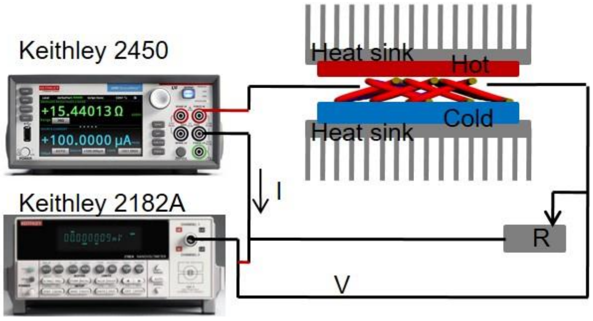

Supplementary Figure 18 | Illustrations of power measurement system. The temperature dependence of power is measured by a homemade system as shown in Supplementary Figure 18 The temperature difference is given by two Peltier elements driven by DC power source. Keithley 2450 is in series with TE textile generator and variable resistor to detect circuit current. Keithley 2182A is employed to obtain output voltage.

Supplementary Tables

Supplementary Table 1 The comparison on power density

References	Power density[a] ($\mu Wm^{-2}K^{-2}$)	Power density[b] ($mWm^{-2}K^{-2}$)
	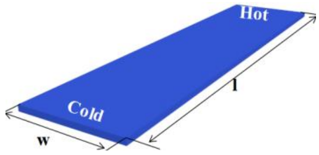	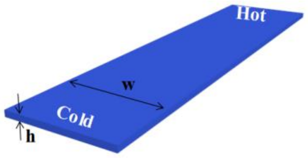
Ref.5 out-of-plane	~0.025	-----
Ref.17 out-of-plane	~0.144	-----
Ref.21 in-plane	~20.74	~2.2
Ref.31 in-plane	~1.1	-----
Ref.32 in-plane	~2.19	-----
Ref.39 in-plane	~3.88	~3.4
Ref.40 in-plane	~0.00259	-----
Ref.41 out-of-plane	~13.33	-----
Ref.42 out-of-plane	~3	-----
Ref.43 in-plane	~0.019	-----
Ref.44 out-of-plane	~6.65	-----
Ref.45 out-of-plane	~2.6	-----
Ref.46 in-plane	~8	~4.2
Ref.47 in-plane	~1.27	~2.5
This work	~35	~180

Here power density[a] is calculated by the output power divided by occupied areas, for film-based 2D devices, occupied area is the product of length, width and numbers of

total TE legs, for our device, the occupied area is the product of length and width of the whole device. The power density[a] marked with red color are calculated by the given output power, occupied areas and temperature difference, those marked with black color are the given value in the listed reported works. Power density[b] is calculated by output power divided by the total cross-sectional area of active TE legs along the heat flow direction. From the point view of practical applications, the power density[a] is compared here.

Supplementary Notes

Supplementary Note 1 Fabrication process of P-hybridized CNTF

CNTF is p-hybridized by dipping into a commercial PEDOT:PSS solution, which is an usual method to enhance the electrical conductivity of CNTF according to previous reports[2]. It is found that infiltration of dopants into the neighboring parts is effectively suppressed due to the high viscosity of PEDOT:PSS. Note that to avoid organic solvent infiltration, dimethyl sulfoxide or ethylene glycol for further doping PEDOT:PSS is not utilized in this paper.

Supplementary Note 2 Thermal design in wearable TE devices

For a thermoelectric generator, electric power (P) converted from heat can be described as $P = \eta Q_{TE}$. Here η is the heat conversion efficiency of a TE harvester, Q_{TE} represents the heat just flowing through TE legs. On the basis of the constant properties approximation in a harvester, the maximum η can be writen as [3,4]

$$\eta_{max} = \frac{\Delta T_{TE}}{T_h} \frac{\sqrt{1+ZT}-1}{\sqrt{1+ZT}+{}^{T_c}/_{T_h}} \tag{1}$$

Here ZT is the thermoelectric *device* figure of merit, which can be approximately

replaced by *material* figure of merit zT according to previous reports[5,6], T_c/T_h is the temperature of cold/hot side, ΔT_{TE} is the temperature difference across TE legs. Refer to $\Delta T_{TE} = T_h - T_c$, equation (1) can be further expressed as

$$\eta_{max} = \frac{\Delta T_{TE}}{T_h} \frac{\sqrt{1+zT}-1}{\sqrt{1+zT}+1-\frac{\Delta T_{TE}}{T_h}} \quad (2)$$

Evidently, the maximum η is related to materials with intrinsic zT, temperature difference across TE legs ΔT_{TE} and temperature of the hot side T_h. $\Delta T_{TE}/T_h$ displayed in equation (2) reprensents the Carnot efficiency η_c[3] ($\eta_c = \Delta T_{TE}/T_h$). Accordingly, the TE generator, as all heat engine, must accept the truth that the heat conversion efficiency η can not be higher than a Carnot efficiency η_c[3]. Thus, η can be extensively described as $\eta=\eta_r\Delta T_{TE}/T_h$, where η_r ($\eta_r < 1$) is the reduced efficiency relative to the Carnot efficiency. In the light of the reported curve[3,7] of η as a function of ΔT_{TE} in the case of a given materials, η is nearly proportional to ΔT_{TE}, indicating an almost constant value of η_r/T_h, in small temperature difference region. In flexible and wearable TE generators harvesting energy from human body, temperature difference is always small. Thus, the efficiency of these generators η can be approximately expressed as[3]

$$\eta = \eta_0 \Delta T_{TE} \quad (3)$$

Here η_0 represents η_r/T_h, which is constant. According to equation (3), electric power P converted from heat for a wearable TE harvester can be described as

$$P = \eta_0 \Delta T_{TE} Q_{TE} \quad (4)$$

On the basis of equation (4), it can be seen that the electricity-generating of a wearable TE devices requires both a ΔT_{TE} and Q_{TE} in the case of given TE materials with intrinsic figure of merit zT.

A simple thermal circuit of a TE harveser is shown as follow[3]:

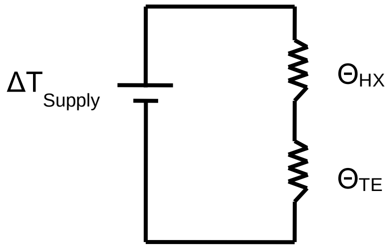

ΔT_{Supply} is the given temperature difference between heat source and environment, Θ_{HX} is the combined thermal resistance of heat exchange in hot and cold side, Θ_{TE} is the thermal resistance of TE materials. According to the above thermal circuit, the heat flowing through TE legs is described by

$$Q_{TE} = \frac{\Delta T_{supply}}{\theta_{HX}+\theta_{TE}} \tag{5}$$

And the ΔT_{TE} across TE legs is described by

$$\Delta T_{TE} = Q_{TE}\theta_{TE} = \frac{\Delta T_{supply}}{\theta_{HX}+\theta_{TE}}\theta_{TE} \tag{6}$$

It implies from equation (5)-(6) that both Q_{TE} and ΔT_{TE} negatively correlates with Θ_{HX}. To optimize the thermal design of TE devices, a minimized thermal resistance of heat exchange is necessary.

Supplementary Note 3 Optimization of power

Thermal resistance of TE materials Θ_{TE} can be expressed as $\Theta_{TE} = 2Nl/\lambda S$ assuming an equal length l, cross-sectional area S and thermal conductivity λ of n/p legs, here N is the total number of TE units. Refer to equation (4)-(6), ΔT_{TE} can be expressed as

$$\Delta T_{TE} = \frac{\Delta T_{supply}}{(\theta_{HX}+kl)}kl \tag{7}$$

And P can be written as

$$P = \eta_0 \frac{\Delta T_{supply}{}^2}{(\theta_{HX}+kl)^2}kl \tag{8}$$

Here $k = 2N/\lambda S$, $l = (L\text{-}4mm)/2$ in our TE devices. Thus, the trend chart of the power

depending on L according to equation (8) can be shown in Supplementary Figure 13, it can been seen that power increases first and then decreases with enhancing L at various temperature differences, and the maximum is obtained at $L = (\Theta_{HX}\lambda S+4N)/N$.

Supplementary References

1 Li, X. *et al.* CsPbX3 Quantum Dots for Lighting and Displays: Room - Temperature Synthesis, Photoluminescence Superiorities, Underlying Origins and White Light - Emitting Diodes. *Adv.Funct.Mater.* **26**, 2435-2445 (2016).

2 Cheng, X. L. *et al.* Design of a Hierarchical Ternary Hybrid for a Fiber-Shaped Asymmetric Supercapacitor with High Volumetric Energy Density. *J. Phys. Chem. C* **120**, 9685-9691 (2016).

3 Priya, S. & Inman, D. J. *Energy Harvesting Technologies: Thermoelectric Energy Harvesting Ch. 11* (Springer. Press, Boston, 2009).

4 Snyder, G. J. & Ursell, T. S. Thermoelectric efficiency and compatibility. *Phys.rev.lett* **91**, 148301 (2003).

5 Nan, K. *et al.* Compliant and stretchable thermoelectric coils for energy harvesting in miniature flexible devices. *Sci. Adv.* **4**, eaau5849 (2018).

6 Snyder, G. J. & Toberer, E. S. Complex thermoelectric materials. *Nat. Mater.* **7**, 105-114 (2008).

7 Zhang, Q. H. *et al.* Realizing a thermoelectric conversion efficiency of 12% in bismuth telluride/skutterudite segmented modules through full-parameter optimization and energy-loss minimized integration. *Energy Environ. Sci.* **10**, 956-963 (2017).
