# Supplementary material for: Stretchable fabric generates electric power from woven thermoelectric fibers
Source: Nat Commun. 2020 Jan 29;11:572. doi: 10.1038/s41467-020-14399-6 (PMC6989526; doi:10.1038/s41467-020-14399-6)
Supplement: Supplementary file 2 — Description of Additional Supplementary Files [file 41467_2020_14399_MOESM2_ESM.pdf]

Description of Additional Supplementary Files

File Name: Supplementary Movie 1
Description: Dynamic detection for the temperature change of TE units that just pass the CNT TE loop through the 3D textiles substrate.

File Name: Supplementary Movie 2
Description: Dynamic detection for the temperature change of TE units directly woven by wrapped CNT TE loop.

File Name: Supplementary Movie 3
Description: Dynamic detection for the temperature change of TE units composed of un-wrapped CNT TE loop.

File Name: Supplementary Movie 4
Description: Demonstration of the good conformality of the TE device.

File Name: Supplementary Movie 5
Description: Dynamic testing process to exhibit the compatibility with body parts of arbitrary geometry or body movement and output stability.
